# Supplementary material for: Daily electrical activity in the master circadian clock of a diurnal mammal
Source: eLife. 2021 Nov 30;10:e68179. doi: 10.7554/eLife.68179 (PMC8631794; doi:10.7554/eLife.68179)
Supplement: Supplementary file 1. — Models were fit to data from seven different neurons, including a non-adapting cell (Figure 5Ai), an adapting-firing cell (Figure 5Aii), an adapting-silent cell (Figure 5Aiii), two Type-A rebound spiking cells (Figures 4 and 6A), and two Type-B delay cells (Figure 6B and O). These cells were chosen for modeling because they are representative of the various responses observed across all the recordings. Each model was fit to data from six voltages traces as illustrated in Figure 4—figure supplement 1. [file elife-68179-supp1.docx]

**Supplementary File 1. Parameter values for the computational models of *Rhabdomys* *pumilio* SCN neurons**. Models were fit to data from 7 different neurons, including a non-adapting cell (Figure 5Ai), an adapting-firing cell (Figure 5Aii), an adapting-silent cell (Figure 5Aiii), two Type-A rebound spiking cells (Figures 4 and 6A), and two Type-B delay cells (Figure 6B and 6O). These cells were chosen for modelling because they are representative of the various responses observed across all the recordings. Each model was fit to data from 6 voltages traces as illustrated in Figure 4-figure supplement 1.

**
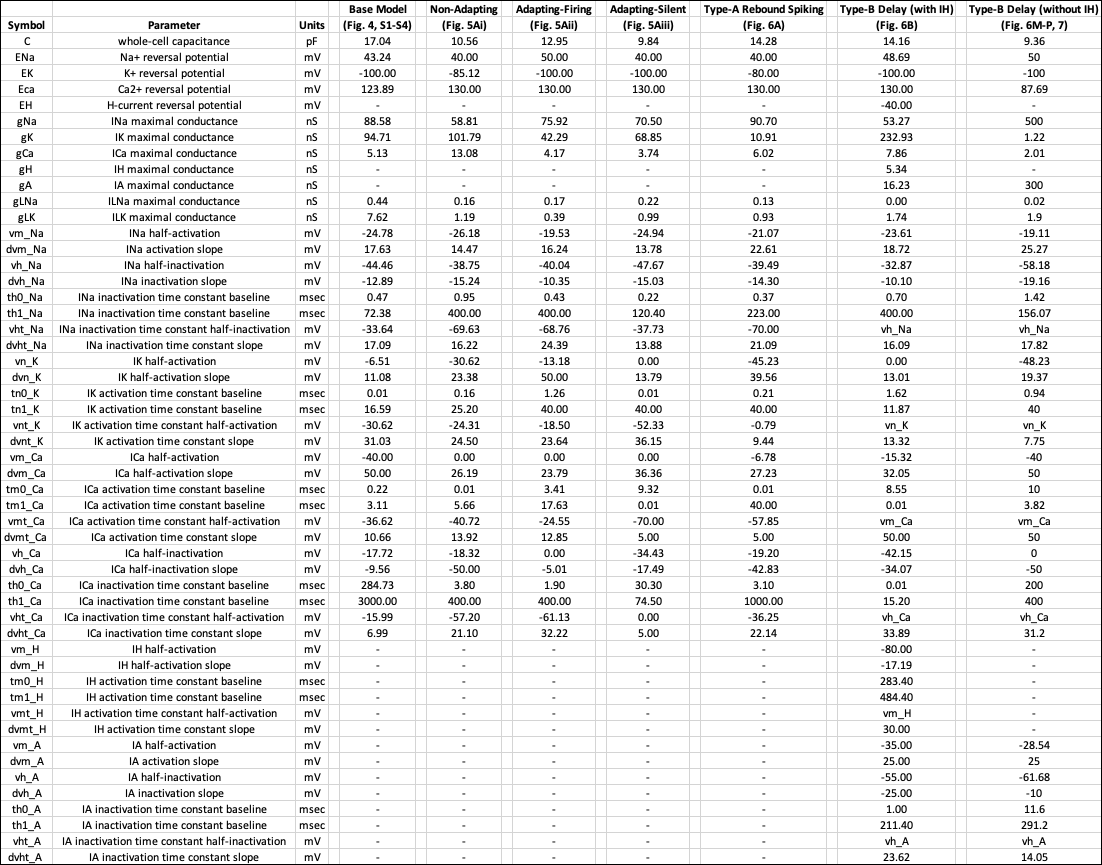
**
